# The Multifunctionality of CD36 in Diabetes Mellitus and Its Complications—Update in Pathogenesis, Treatment and Monitoring

**DOI:** 10.3390/cells9081877

**Published:** 2020-08-11

**Authors:** Kamila Puchałowicz, Monika Ewa Rać

**Affiliations:** Department of Biochemistry, Pomeranian Medical University, 70-111 Szczecin, Poland; carmon12@gmail.com

**Keywords:** cardiomyopathy, hyperglycemia, fatty acids, inflammation, insulin resistance, lipotoxicity, nephropathy, neuropathy, oxidative stress, retinopathy

## Abstract

CD36 is a multiligand receptor contributing to glucose and lipid metabolism, immune response, inflammation, thrombosis, and fibrosis. A wide range of tissue expression includes cells sensitive to metabolic abnormalities associated with metabolic syndrome and diabetes mellitus (DM), such as monocytes and macrophages, epithelial cells, adipocytes, hepatocytes, skeletal and cardiac myocytes, pancreatic β-cells, kidney glomeruli and tubules cells, pericytes and pigment epithelium cells of the retina, and Schwann cells. These features make CD36 an important component of the pathogenesis of DM and its complications, but also a promising target in the treatment of these disorders. The detrimental effects of CD36 signaling are mediated by the uptake of fatty acids and modified lipoproteins, deposition of lipids and their lipotoxicity, alterations in insulin response and the utilization of energy substrates, oxidative stress, inflammation, apoptosis, and fibrosis leading to the progressive, often irreversible organ dysfunction. This review summarizes the extensive knowledge of the contribution of CD36 to DM and its complications, including nephropathy, retinopathy, peripheral neuropathy, and cardiomyopathy.

## 1. Introduction

Widespread research aimed to thoroughly understand the cluster of differentiation 36 (CD36) has been carried out for the last 40 years. At first, it was focused on the characteristics of the CD36 gene and protein, its tissue and subcellular localization, and its function, and at a later stage also understanding the role of CD36 in the pathogenesis of many diseases. The role of CD36 in infection of *Plasmodium falciparum* [1] and atherosclerosis and cardiovascular disease [2] has attracted the most interest; however, more recently there has been an increased focus on its role in diabetes mellitus (DM). There are several explanations for this. First, DM has become a major global public health problem due to its high prevalence and serious health effects resulting from its numerous complications, such as premature atherosclerosis, nephropathy, retinopathy, neuropathy, and cardiomyopathy. DM and its complications significantly reduce the quality of life of patients, and are associated with an increase in mortality [3,4]. Second, CD36 is expressed in many cells sensitive to metabolic abnormalities related to metabolic syndrome, prediabetes, and DM. Third, CD36 function is associated with the modulation of the inflammatory response and carbohydrate and lipid metabolism of these cells. This made CD36 a serious “candidate” for another protein involved in the pathogenesis of DM and its complications. 

CD36 is important in prediabetes when it participates in the development of insulin resistance in adipose tissue [5,6], the liver [7], skeletal muscles [8,9], and the heart [10]. CD36 also mediates both pancreatic β-cell dysfunction and β-cell mass reduction [11,12], contributing to the reduction of insulin secretion and progression to DM [13]. Conditions such as hyperglycemia and dyslipidemia significantly change CD36 expression, its function, and its signaling pathways. The CD36-dependent mechanisms are important events in the pathogenesis of diabetic complications, such as nephropathy [14,15,16,17], retinopathy [18,19,20], neuropathy [21,22], and cardiomyopathy [23,24,25,26]. Importantly, CD36 disrupts the functions of organs in a tissue-specific manner by several different mechanisms. The abnormalities of carbohydrate and lipid metabolism correlate with changes to CD36 expression and subcellular localization [8,10,14,15,16,25,27,28,29,30,31,32] and contribute to an increase in uptake of fatty acids (FAs) and modified lipoproteins; intracellular accumulation of lipids such as triacylglycerols (TAGs), diacylglycerols (DAGs), and ceramides (CERs); and oxidative and endoplasmic reticulum (ER) stress—and thus activation of a number of signaling pathways that initiate inflammation, modulate insulin response and the utilization of energy substrates, and stimulate cell death and fibrosis leading to progressive, often irreversible organ dysfunction [5,7,9,10,11,14,15,16,18,19,22,23,26].

In this review, we have summarized the latest reports on the roles of CD36 in the pathogenesis of DM and its complications. Particular attention was paid to (1) the characteristics of CD36; (2) the role of CD36 in the pathogenesis of DM and its complications, including nephropathy, retinopathy, neuropathy, and cardiomyopathy; (3) the possibility of using CD36 as a therapeutic target; and (4) plasma soluble CD36 (sCD36) as a marker of DM and related diseases. Our considerations may form the basis for the development of further research and a new approach to treatment and new diagnostic or prognostic markers for DM and its complications.

## 2. Characterization of CD36

CD36 is a multifunctional transmembrane glycoprotein receptor that belongs to the class B scavenger receptor family. It is alternatively known as leukocyte differentiation antigen CD36, platelet glycoprotein IV (GPIV), glycoprotein IIIb (GPIIIb), PAS-4 protein (PAS IV), or fatty acid translocase (FAT) [33]. The discovery of CD36 was due to Kobylka and Carraway [34], who in 1973 demonstrated the presence of a membrane protein in breast epithelial cells that could not be hydrolyzed in milk fat globules. In 1977 this new protein was isolated from platelets and identified as GPIV by Clemetson et al. [35]. Then Tandon et al. characterized GPIV and found its structural similarity with leukocyte differentiation antigen CD36 [36,37].

Human CD36 is encoded by *CD36* located on chromosome 7q21.11 [38]. The structural organization of human *CD36* was described by Armesilla et al. [39,40]. The gene encodes a protein of 472 amino acids with a molecular weight of 78–88 kDa depending on cell type and degree of glycosylation [41]. It has a hairpin-like structure and contains two transmembrane domains, a large highly glycosylated extracellular loop containing ligand-binding sites, and two short intracellular domains at C and N terminals [42]. Various molecular mechanisms for regulating *CD36* gene expression [43,44,45] and posttranslational modifications [42] are responsible for the multiplicity of interactions and functional diversity of CD36. The regulatory mechanisms of the *CD36* gene transcription involves interactions with several transcriptional factors: CCAAT/enhancer-binding protein (C/EBP) [46], peroxisome proliferator-activated receptor (PPAR) [47], and activating transcription factor 2 (ATF2) [48]. Tissue-specific posttranslational modifications, such as glycosylation, palmitoylation, acetylation, or phosphorylation might modulate CD36 cellular location and ligand binding [42,49].

Many types of cells express CD36: platelets [37], erythrocytes [50], monocytes and macrophages [51], microvascular endothelial cells [52], adipocytes [53], skeletal and cardiac muscle cells [54], islets of Langerhans [12], kidney cells [55], retina cells [20], and peripheral nerve cells [21]. There is also a circulating form of CD36 called soluble CD36 (sCD36). The mechanism of sCD36 formation is not entirely clear. It was initially thought that sCD36 is part of the extracellular segment of CD36 cleaved by a plasma protease [56]; however, a new report indicates that sCD36 is a full-length protein associated with a subset of circulating microparticles, which are small (0.1–1 μm in diameter) membranous microvesicles [57,58]. They are shed from cell membranes as a result of cell activation, apoptosis, and senescence—by, for instance, platelets, erythrocytes, leukocytes, and endothelial cells. This finding may facilitate identification of the cellular source of sCD36 in diabetic patients.

CD36 is a receptor for a broad range of ligands and mediating various signaling pathways depending on the cell type, but usually, signal transduction is initiated via Src family kinases and extracellular signal-regulated kinases (ERKs) [2,46]. Some ligands are proteinaceous, such as thrombospondin (TSP) [51], collagen [37], amyloid β [59], growth hormone-releasing peptides (GHRP) [47], and advanced glycation end products (AGEs) [60], while others are lipidic, such as long-chain FAs [53,54], or both proteinaceous and lipidic, for instance, oxLDL [61] and microbial diacylated lipopeptides [62]. Apoptotic cells can also act as a ligand for CD36 [63]. Many of these ligands play important roles in the pathogenesis of DM and its complications. 

## 3. Diabetes Mellitus—Is It an Epidemic?

DM is a group of chronic metabolic diseases characterized by persistently increased blood glucose levels [64] resulting from defects in insulin secretion, loss of insulin responsiveness, or both [65]. Commonly DM is classified into two main types, type 1 (T1DM) and type 2 (T2DM). Due to the high heterogeneity of DM, a new subclassification in adult-onset diabetes patients was proposed in 2018 [66]. This includes five clusters classified based on patient characteristics (glutamate decarboxylase antibodies, age at diagnosis, BMI, glycated hemoglobin (HbA1c), and homeostatic model assessment two estimates of β-cell function and insulin resistance) and risk of diabetic complications. The advantages of clustering are the identification of patients with high risks of complications and obtaining information about underlying disease mechanisms, and thus supporting choice of therapy. DM has become a major public health problem that is approaching epidemic proportions globally. According to the International Diabetes Federation (IDF) [67] the world prevalence of DM among adults reached 451 million in 2017, and about five million deaths worldwide were attributable to DM. DM presents a large global burden for social, financial, and health systems.

Various factors, such as family history, race, ethnic background, age, obesity, insulin resistance, sedentary lifestyle, polycystic ovary syndrome, and diseases of the pancreas can result in losing the control of blood glucose and the development of prediabetes (impaired glucose tolerance or impaired fasting glucose) which progresses to DM. DM manifests clinically as hyperglycemia defined as excessively high levels of glucose in the blood (fasting blood glucose ≥ 126 mg/dL, 2 h plasma glucose ≥ 200 mg/dL during oral glucose tolerance test, HbA1c ≥ 6.5% or random plasma glucose ≥ 200 mg/dL) [64].

DM-related chronic hyperglycemia and dyslipidemia are associated with the development of complications resulting from progressive damage of different organs, particularly the kidneys, eyes, peripheral nerves, heart, and blood vessels [4]. Microvascular lesions, such as nephropathy and retinopathy, are initiated with hyperglycemia, while atherosclerotic macrovascular lesions develop as early as in prediabetes. The atherosclerotic process is much more aggressive in diabetics than non-DM patients. Therefore, they are at a higher risk of developing cardiovascular disease and ischemic events [68]. The complications of DM, especially premature atherosclerosis, cardiomyopathy, and nephropathy, are responsible for increased morbidity and mortality risks in diabetics [4].

## 4. Association of sCD36 with Diabetes Mellitus

The association of plasma sCD36 concentration with metabolic syndrome and DM has been studied by many researchers. Plasma sCD36 was first identified as a potential novel biomarker for T2DM by Handberg et al. [69]. They indicated the association between concentration of sCD36 and glycemia measured as both fasting glucose and HbA1C. The role of sCD36 as a biomarker for T2DM was later confirmed by other studies [70,71]. Koonen et al. [72] summarized that sCD36 reflects tissue CD36 expression level, and is derived mainly from monocytes and macrophages. T2DM related to factors such as insulin resistance, high oxLDL, systemic low-grade inflammation, or hepatosteatosis, stimulates CD36 expression in monocytes and macrophages localized in adipose tissue, the liver, and arteries leading to elevated plasma sCD36. Activated platelets are another indicated source of sCD36, which is argued by a partial reduction in sCD36 levels after treatment with low-dose aspirin [73]. However, the origin of sCD36 and the question of whether the concentrations of sCD36 reflect CD36 tissue expression or activity of CD36-mediated signaling pathways are currently discussed.

There are studies that deny the existence of the association of sCD36 with DM. Castelblanco et al. [74] assumed as a primary study objective evaluating the potential association of sCD36 with DM. They did not show the differences in plasma sCD36 levels between patients with T1DM or T2DM and non-diabetic control participants. All subjects had normal renal function and were not burdened with advanced late diabetic complications. This may suggest that hyperglycemia is not one of the main factors contributing to plasma sCD36 levels in patients with DM. An increase in sCD36 concentration was only observed in patients with T2DM who had dyslipidemia or used antiplatelet drugs.

The authors reported several potential causes of non-compliance between studies. One is that sCD36 appears to be dependent on risk factors of cardiovascular disease and T2DM. There is an association between sCD36 level and cardiovascular disease risk factors such as hypertension, dyslipidaemia, tobacco exposure, age, sex, and BMI [74]. Many investigators have shown an association of sCD36 with components of the metabolic syndrome, such as insulin resistance and central obesity [69,75,76,77], which are key predictors of T2DM and risk factors of accelerated atherosclerosis in this group of patients. However, there was a study on the middle-aged patient population with T2DM from Jordan, in which no difference in sCD36 levels was found between patients with metabolic syndrome and healthy controls [78]. The association with the risk of T2DM has also been described, but it was not independent of fasting glucose, insulin, or adiposity [76,79]. The sCD36 index proposed by Kim et al. [80] seems to be a better marker of T2DM risk in the general population than the concentration of sCD36. It is calculated with concentrations of sCD36 and fasting glucose in plasma and is independent of other risk factors.

Furthermore, differences between the characteristics of the studied populations are inevitable. They include the size of the population, age, time of duration of DM, components of the metabolic syndrome, occurrence of diabetic complications, and the drugs used. It is possible that the factors specific for the population may affect sCD36 concentrations and thus this needs to be checked. Among diabetic drugs, the relationship between the sCD36 levels and the use of statins and antiplatelet drugs was evaluated. Chmielewski et al. [81] showed a positive correlation of sCD36 level with statin use, but Castelblanco et al. [74] denied it. There is also no unequivocal position as to the effect of antiplatelet drugs. Both a reduction [73] and an increase [74] in sCD36 levels after antiplatelet drugs were observed. However, in the first case low doses of aspirin were used, and in the second one the authors did not specify which antiplatelet drugs were used.

In the longer term, an important issue is to establish a well-standardized method for the evaluation of circulating CD36. sCD36 is not truly soluble but is CD36 protein associated with microparticles (CD36+MPs) [57]. In healthy individuals, more than 90% of circulating microparticles in plasma are platelet-derived. However, various pathological conditions, such as metabolic syndrome, T2DM, and cardiovascular disease, may change the cellular source and the amount of microparticles in plasma [82]. It was observed that the levels of circulating microparticles of various origins (platelets, monocytes, endothelium cells), except for leukocytic ones, are significantly higher in patients with T2DM than in healthy individuals [58]. Alkhatatbeh et al. [82] indicated that concentrations of CD36+MPs in obese patients with T2DM were higher compared to lean and obese controls. The main cellular source of CD36+MPs in patients with T2DM were erythrocytes, but in controls the main source was endothelial cells. Moreover, they confirmed that flow cytometric analysis of plasma CD36+MPs levels was a much better biomarker for DM than plasma CD36 protein levels determined by ELISA. However, the authors pointed out in their other study that standardized ELISA which could be performed in any basic laboratory would be more favorable for common measurements of a biomarker than the specialized flow cytometry analysis [78]. Therefore, research should be performed to establish a well-standardized method for determining circulating CD36, including plasma preparation. This will provide comparable sCD36 results between different study groups. Taken together, it is not clear whether the observed increased level of sCD36 is a direct result of DM or the metabolic disorders underlying DM. The presented findings rather suggest that sCD36 level is the outcome of pathogenic effects of various DM risk factors, such as components of metabolic syndrome, and its complications, but not DM per se. Moreover, it cannot be ruled out that the measurement of sCD36 or CD36+MPs may be a helpful tool in monitoring the development of DM, but the method of circulating CD36 measurement requires standardization.

## 5. The Role of CD36 in the Pathogenesis of DM

The mechanism underlying the positive association between the sCD36 and T2DM is not clearly defined, but current reports implicate that CD36 contributes to the development of T2DM on two levels: insulin resistance [5,7,8,10] and pancreatic β-cell dysfunction and damage [11,12]. Insulin resistance and defects in pancreatic β-cell function are major pathophysiologic events in the pathogenesis of T2DM [13]. Increased sCD36 levels were observed in prediabetes, e.g., impaired glucose tolerance [75,83] and polycystic ovary syndrome [84]. It is likely that sCD36 level reflects early changes in surface CD36 expression in some tissues as a result of developing insulin resistance [76]. The causes for the increased sCD36 in DM are cellular activation, to a lesser extent apoptosis [58], but probably also senescence. Increased cell senescence has been demonstrated in many tissues affected by DM, for instance in the skin, pancreas, adipose tissue, kidney, retina, the peripheral nerve and cardiovascular system [85,86], and is a stimulator of increased microvesicle release [87]. The role of CD36 in the pathogenesis of DM is also supported by other studies. In non-diabetic patients, the concentration of sCD36 in plasma was highly related to insulin resistance, carotid atherosclerosis and fatty liver [77], whereas lifestyle and surgical interventions leading to a weight loss result in a decrease in sCD36 levels. Weight loss in obese children was associated with a decrease in sCD36 levels corresponding with the improvement of insulin resistance, lipid profile and liver fat [88]. sCD36 decline after bariatric surgery in morbidly obese individuals was accompanied by the improvements in fat distribution and ectopic hepatic fat accumulation [89]. Hence the conclusion that CD36 is associated with many components of the metabolic syndrome.

### 5.1. Insulin Resistance

Insulin resistance is one of the metabolic defects underlying prediabetes and T2DM. The risk factors of developing insulin resistance are, among other things, obesity and high-fat diet. The pathogenesis of insulin resistance is very complex. Currently, researchers are focusing on several components, including inflammation, oxidative stress, and excess lipid accumulation that can lead to reduced insulin sensitivity in the adipose tissue, the liver, the heart, or muscles with abnormal metabolism of glucose and lipids [90]. Insulin resistance with impaired glucose metabolism induced by a high-fat diet appears faster in adipose tissue and the liver than in muscles in which the deterioration of insulin action requires more time. It is associated with the sensitivity of tissues to the effects of dietary lipid overload [91,92]. The role of CD36 in the pathomechanism of insulin resistance in particular tissues has been demonstrated by many researchers and requires a separate discussion. Generally, the mechanism of insulin resistance results from defective insulin action at target cells. However, insulin has different functions depending on the cell types; therefore, the functional consequences of insulin resistance differ between the target tissues [93]. It seems that the contribution of CD36 is to promote post-receptor defects, not changes in the function of the insulin receptor itself. The alterations in lipid metabolism upon FA oversupply mediated by CD36 are particularly important for the development of insulin resistance.

#### 5.1.1. Adipose Tissue

The role of CD36 in adipocytes is to mediate both the storage and mobilization of energy by the uptake and release of FAs in response to lipolytic stimuli [94,95]. The hypothesis about the involvement of CD36 in the insulin resistance in adipose tissue was initiated by the observation of increased CD36 expression associated with obesity in a rodent model [96,97] and humans [28]; moreover, the analysis of transcriptional regulation of CD36 in obesity supports it [98]. CD36 expression in subcutaneous adipose tissue (SAT) appears to be more sensitive to metabolic disturbances compared to visceral adipose tissue (VAT) [28]. In general, accumulation of visceral intra-abdominal adipose tissue (central obesity) is associated with insulin resistance and increased risk of metabolic disease, whereas accumulation of SAT (fat in the hips and flanks) has no adverse effect and may even be protective against metabolic syndrome [99]. However, deep SAT, one of the abdominal white adipose tissue depots, differ metabolically from superficial SAT and is closely related to the pathophysiology of obesity complications by increasing inflammation and oxidative stress [100]. SAT CD36 expression was upregulated in patients with obesity and T2DM, whereas in VAT it was not different in lean, overweight, and obese participant and was only increased in T2DM. CD36 expression was strongly associated with BMI, and glucose and insulin plasma concentrations. Hyperinsulinemia is a potential factor inducing CD36 expression in this situation [28]. In rodent models, it was confirmed that CD36 underlies the progression of obesity-associated metabolic dysfunctions, such as visceral fat accumulation with impairment in glucose clearance and abnormal fasting blood glucose level. CD36 inhibition may prevent visceral obesity and improve insulin resistance [97]. Reduced adiposity is a feature of *CD36^−/−^* mice [101]. Interestingly, CD36 mRNA and protein expression in adipose tissue was negatively correlated with intrahepatic TAG content presented as a marker of the metabolic disorders associated with obesity [102]. We suppose that this is the result of internalization of adipocytic CD36 in response to excess FA generated during adipose lipolysis typical for insulin resistance. CD36 internalization stimulated by products of lipolysis, FAs, was identified as a novel negative feedback mechanism regulating stimulation of lipolysis in adipocytes [94]. The decrease in CD36 expression is accompanied by a decrease in the production of adiponectin and its plasma concentration. Low adiponectin level is associated with hepatic and skeletal muscle insulin resistance [102]. The presented studies indicate that obesity increases CD36 expression in adipose tissue, which is associated with the stimulation of lipolysis and the development of insulin resistance. However, persistently increased lipolysis in adipose tissue may reduce CD36 expression, predisposing one to lowered adiponectin production and insulin resistance in liver and muscles.

One of the mechanisms associated with obesity and responsible for the decrease of insulin sensitivity in white adipose tissue (WAT), but also in liver and skeletal muscle, is systemic chronic low-grade inflammation [103]. CD36 is a regulator of adipose tissue inflammation. Effectors of inflammation are macrophages infiltrating adipose tissue in obesity, but also adipocytes themselves. The key event initiating the infiltration of macrophages to WAT is the CD36-dependent inflammatory response and apoptosis of adipocytes in response to diet-induced obesity. An inflammatory response in macrophages is also dependent on CD36. The exact intracellular mechanisms are not known, but CD36 has been reported to promote inflammation by the alteration of lipid metabolism, resulting in ER stress and the activation of stress signaling pathways, such as the c-Jun N-terminal kinase (JNK) and nuclear factor kappa-light-chain-enhancer of activated B cells (NFκB) cascades. It is clear that CD36 regulates proinflammatory cytokine production from both adipocytes and macrophages, enhancing inflammation in adipose tissue in a synergistic manner. It favors adipose tissue remodeling and expansion [6]. Salvianolic acid B, a CD36 antagonist, counteracts obesity-induced macrophage infiltration and inflammation in adipose and hepatic tissues [97]. Moreover, CD36 mediates a proinflammatory signaling loop between adipocytes and macrophages, which promotes insulin resistance in response to hyperlipidaemia. OxLDL disrupts insulin signaling in adipocytes and macrophages by interaction with CD36 associated with Fyn and Lyn tyrosine kinases, and then the activation of JNK, which leads to phosphorylation of insulin receptor substrate 1 (IRS-1) on serine 307, resulting in uncoupling of IRS-1 from the insulin receptor and decreasing of IRS-1 tyrosine phosphorylation at sites necessary for the interaction with phosphoinositide 3-kinase (PI3K), thereby impairing insulin action. Induced metabolic abnormalities include impaired glucose uptake, decreased secretion of adiponectin, and increased lipolysis [5].

As already mentioned, most known mechanisms of insulin resistance in adipose tissue are associated with autocrine or paracrine release of inflammatory cytokines which may impair insulin signaling. However, the fact that early resistance to inhibition of lipolysis by insulin precedes significant inflammation suggests a mechanism independent of inflammation as the initial defect in adipose tissue insulin resistance [93]. There is a report that CD36 is required for optimal stimulation of adrenergic signaling-activated lipolysis via cAMP turnover and protein kinase A (PKA) activation [94]. On the other hand, CD36 appears to limit inhibition of lipolysis by insulin. In CD36-deficient mice, insulin was more potent in inhibiting lipolysis, probably due to fewer hypertrophic adipocytes. Furthermore, a high-fat diet induces significantly less inflammation in adipose tissue of these mice. These results indicate higher insulin sensitivity of the adipocytes in *CD36^−/−^* mice [101]. It is unclear, however, whether the participation of CD36 in the mechanism of insulin resistance in adipose tissue relies more on modulating lipid storage or directly to lipolysis.

Additionally, metabolic crosstalk between adipocytes and the extracellular matrix (ECM) affects glucose metabolism in adipose tissue. AGEs’ interaction with the ECM reduces glucose uptake and favors the development of insulin resistance. CD36 is a receptor for AGEs, but it is not involved in this pathomechanism [104]. However, the interaction of AGE, glycolaldehyde-modified BSA (GA-BSA) or oxLDL with CD36 downregulates leptin expression in adipocytes [105,106] which is associated with ectopic fat deposition and lipotoxicity [107]. It also reduces insulin sensitivity of adipose tissue in metabolic syndrome [105,106].

**Figure 1:**
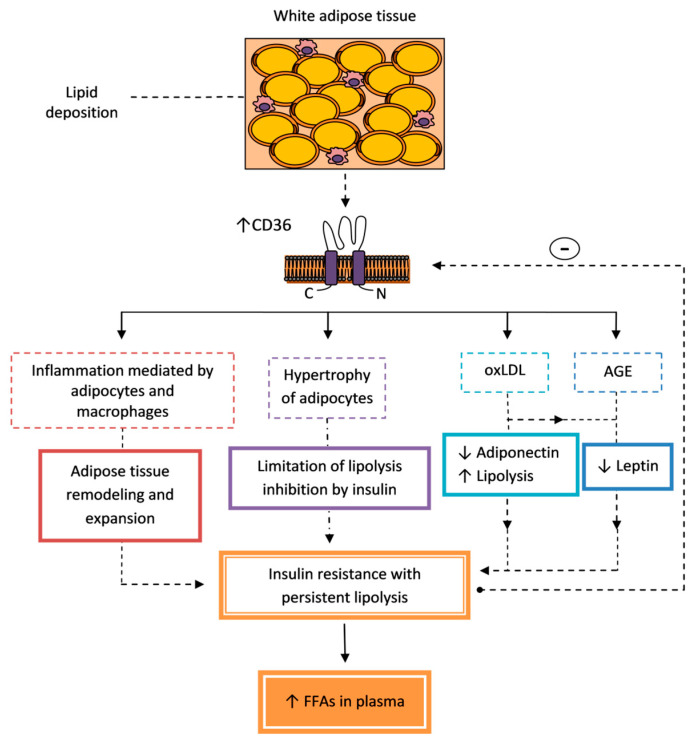
The contribution of CD36 to the pathogenesis of insulin resistance in adipose tissue. The roles of CD36 in the reduction of adipose tissue insulin sensitivity with increased lipolysis are to promote inflammation and promote remodeling of the adipose tissue with hypertrophy of adipocytes, and an adverse profile of adipokines mediated by oxidized low-density lipoprotein (oxLDL) and advanced glycation end product (AGE). Ultimately, this leads to a significant release of fatty acids (FAs) and an increase in free fatty acid (FFA) concentration in plasma, exceeding the energy needs of such organs as the liver, muscle, and heart.

The contribution of CD36 to the pathogenesis of insulin resistance in adipose tissue is summarized in Figure 1.

It should be noted that WAT is the tissue that first responds to excess nutrients, which makes it the most sensitive to the development of insulin resistance. The characteristic features of insulin resistance in adipose tissue are intensified lipolysis (normally inhibited by insulin) and an increase in free fatty acids (FFAs) in the plasma [91]. Therefore, the role of CD36 in long-chain FA uptake into cardiac and skeletal myocytes is particularly important in insulin resistance pathogenesis. CD36 appears to be the predominant membrane protein transporting FAs in enterocytes, adipose tissue, skeletal muscles, and the heart. Less significant membrane proteins facilitating FA transport are fatty acid-binding protein (FABP_pm_), fatty acid transport proteins 1–6 (FATP1-6), and caveolin-1 [108]. Importantly, FA transport through endothelial cells is equally important as the transport through parenchymal cell membranes because it limits tissue FA uptake [109]. The regulation of FA uptake involves the upregulation of CD36 gene expression by binding FAs to PPAR (long-term) and the subcellular vesicular recycling of CD36 from endosomes to the plasma membrane (short-term) [44]. The *CD36* gene promoter contains peroxisome-proliferator-activated-receptor responsive elements (PPRE) regulated by PPARα or PPARγ, for instance, in macrophages [110,111], cardiac microvascular endothelial cells [112], smooth muscle cell [113], cardiac and skeletal myocytes [108], and HK-2 renal tubule epithelial cells [15]. Excessive plasma FFAs, derived from lipolysis of adipose tissue or diet, may result in the intracellular influx of FAs exceeding the capacity of cells for their utilization. CD36 plays a key role in this process leading to pathological accumulation of lipids (TAGs, DAGs and CERs) in non-adipose organs, such as liver, heart and muscle (ectopic lipid accumulation), and initiating lipotoxicity and insulin resistance [98,108]. Additionally, macrophages of adipose tissue can take up the released FAs and store them as TAG droplets without activation of inflammatory pathways [114]. However, it is a buffering mechanism protecting metabolic tissues from the damage caused by ectopic accumulation of saturated FAs [115].

#### 5.1.2. Liver

The liver is a major recipient of FAs because of its very high capacity to take up plasma FAs. CD36 expression in the liver is relatively low [108] and it is not a major facilitator of FA uptake in a normal liver [101]. However, CD36 can be highly upregulated in a response to the elevated plasma FFA level to contribute to lipid accumulation and activation of inflammation [7,29]. Prolonged sucrose consumption [116,117] and hyperinsulinemia [30] also cause CD36 upregulation in the liver. CD36 overexpression is induced by liver X receptor (LXR), pregnane X receptor (PXR), and PPAR-γ [30,118]. An increased amount of CD36 in the hepatocytes’ plasma membrane was also observed in obese rats in association with its translocation induced by hyperinsulinemia [119]. CD36 in the liver might play dual roles: facilitating FA flux under conditions of high FA supply and transducing intracellular signals that regulate phospholipids deacylation and eicosanoid production, processes that influence assembly and secretion of a very-low-density lipoprotein (VLDL) [120].

CD36 was found to be directly linked to the development of hepatosteatosis under conditions of elevated FFAs or hyperinsulinemia. Moreover, the effect of inappropriate FA flux to the liver is a critical stimulation of VLDL secretion with limitation of insulin’s ability to inhibit this process [107]. The levels of intrahepatic lipids and insulin resistance are positively correlated with sCD36 level. Hepatocytes are a potential source of sCD36 in patients with non-alcoholic fatty liver disease (NAFLD) [121]. Deletion of *CD36* in a mouse model fed with a high-fat diet improved steatosis and whole-body insulin sensitivity by lowering FA uptake; lowering the accumulation of TAGs, DAGs, and cholesterol esters; and reducing inflammation [7].

It is well established that ectopic fat deposition in the liver contributes to the development of hepatic insulin resistance [7]. Cellular accumulation of TAGs per se does not initially damage hepatocytes and protects cells from lipotoxicity [122], whereas accumulated bioactive lipids, such as CERs and DAGs, are associated with the development of hepatic insulin resistance in hepatosteatosis [123,124]. When the energy needs of the hepatocytes are met and their capacity of energy storage is full, FAs start to couple to other substrates, sphingoid backbones, rather than the glycerol resulting in accumulation of CERs [124]. Prolonged CER accumulation favors insulin resistance. Among the most important action mechanisms of CERs associated with insulin resistance of various tissues are: antagonizing insulin signaling, which leads to the impairment of glucose uptake or storage nutrients such as TAGs and glycogen; activation of inflammatory pathways; stimulation of FA uptake; inhibition of FA oxidation; and induction of apoptosis [123]. In the liver these effects include increases in FA uptake (via CD36) and synthesis of TAGs (via SREBP), and alterations of mitochondrial functions (increased mitochondrial fission and decreased efficiency), which predispose one to hepatosteatosis [124]. Accumulated CERs may intensify steatosis by the activation of the protein kinase C ζ (PKCζ) pathway resulting in CD36 translocation to the plasma membrane and an increase FA uptake [125]. Replacing CERs with dihydroceramides eliminates this effect and protects people from hepatic steatosis [126]. However, precursors of TAGs and DAGs are associated with hepatic insulin resistance through the induction of protein kinase Cε, which results in the inhibition of insulin signaling [127].

**Figure 2:**
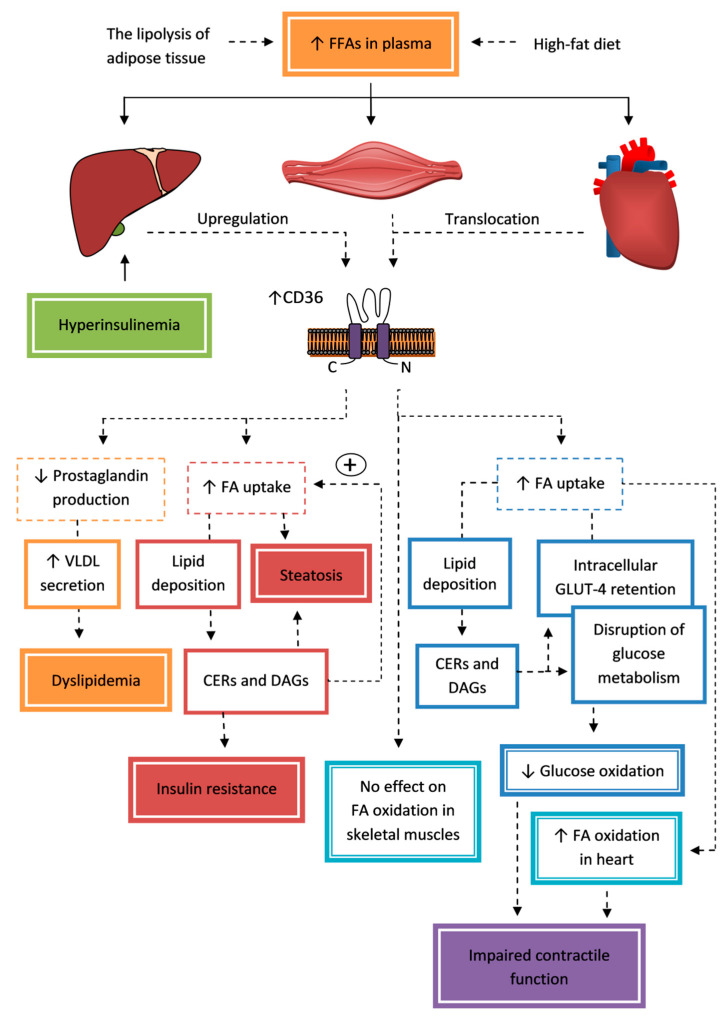
The contribution of CD36 to the pathogenesis of insulin resistance in the liver, muscle, and the heart. Increased free fatty acid (FFA) concentrations in plasma are a factor initiating upregulation and translocation of CD36 with an increase in this transporter expression in the plasma membrane for the liver and myocytes. This leads to an increase in fatty acid (FA) influx via CD36 with pathological accumulation of triacylglycerols (TAGs), diacylglycerols (DAGs), and ceramides (CERs) initiating lipotoxicity and insulin resistance. A typical feature of an insulin-resistant liver is steatosis, in which accumulating lipids antagonize insulin signaling, re-increase CD36 expression in the cell membrane, disrupt mitochondrial function, and increase TAG accumulation. However, increased expression of CD36 in hepatocytes also stimulates the removal of FAs by very-low-density lipoprotein (VLDL) secretion, but this is associated with the worsening of dyslipidemia. Skeletal and cardiac myocyte overexposure to lipids results in glucose transporter type 4 (GLUT-4) being entrapped in non-endosomal storage and the translocation of CD36 to the sarcolemma associated with excessive FA transport and lipid accumulation interfering insulin signaling by a decreased glucose transport and its incorporation to glycogen. Moreover, in cardiomyocytes, FA oversupply changes the expression of genes associated with FA and glucose utilization. Ultimately, these actions result in a transition to energy from FAs instead of glucose and contractile dysfunction.

Moreover, insulin resistance in hepatosteatosis is associated with a dysfunction of glycogen synthesis. In contrast, increased glycogen synthesis in the liver improves glucose tolerance independently of insulin signaling [128]. A study in mice overexpressing CD36 in the liver indicated stimulation of glycogen synthesis, probably by inhibition of prostaglandin formation, which protected from fasting hypoglycaemia and improved glucose tolerance and insulin sensitivity. Furthermore, they observed that CD36 signaling may be helpful in preventing fatty liver by increasing the formation and secretion of VLDL. They proposed that CD36 plays a protective role in lipid overload and metabolic stress caused by a high-fat diet and prolonged fasting [129]. Nassir et al. [120] confirmed that CD36 plays a role in VLDL secretion mediated at least in part by decreasing prostaglandin production. They suggest that the increased CD36 expression in NAFLD might be in part an adaptation to conditions of increased lipid accumulation by increased VLDL secretion. However, the authors pointed out that the impact of CD36 on hepatosteatosis might differ depending on metabolic situation. They think that CD36 deletion is protective for steatosis when the major cause is excessive FA uptake (high-fat diet), but might intensify lipid accumulation when hepatic lipogenesis is a major contributor to steatosis. Another reason for the described inconsistencies in the perception of the role of CD36 is most likely the use different animal models of steatosis (other rodent species; genetic modifications or factors initiating steatosis). However, researchers rather indicate the association of CD36 with pathogenesis of hepatosteatosis and liver insulin resistance. The contribution of CD36 to the pathogenesis of insulin resistance in the liver is summarized in Figure 2.

#### 5.1.3. Skeletal and Cardiac Muscles

CD36 is known to suppress adenosine monophosphate-activated protein kinase (AMPK) when FA availability is low, keeping it quiescent, while FA binding to CD36 during fasting (when FAs are at their highest availability) limits this suppression. Linking AMPK activation to FA availability is important for the maintenance of cellular FA homeostasis. Actions of CD36 are through the modulation of a CD36/Fyn/LKB1/AMPK protein complex. The activation of AMPK response to FA supply promotes recruitment of CD36 to the plasma membrane and β-oxidation of FAs by the reduction of the β-oxidation inhibitor, malonyl-CoA [130]. The translocation of CD36 to the plasma membrane is dependent on activation of AMPK which is induced by leptin and is required for its action [131]. CD36 translocates to the mitochondria in response to muscle contraction, where it participates in the upregulation of FA oxidation by metabolic stimuli [132]. CD36 is also important for optimal insulin stimulation of glucose metabolism in the postprandial period. CD36 interacts with the insulin receptor, thereby promoting tyrosine phosphorylation of the receptor by Fyn kinase and enhancing downstream signaling, causing glucose uptake and utilization. In contrast, the presence of saturated FAs, but not unsaturated FAs, inhibits CD36-Fyn-dependent phosphorylation of the insulin receptor [133]. Therefore, CD36 influence on muscle fuel choice between FA and glucose depends on the metabolic state. The dysregulation of the CD36-dependent pathways of FA and glucose metabolism in skeletal muscles, occurring in chronic excess of FA and DM, may reduce their ability to modulate FA, and glucose utilization depends on energy needs, which will result in lipid accumulation and insulin resistance [130,133].

Increased delivery of FFAs to the heart or skeletal muscles results in an imbalance in the subcellular recycling of CD36. Compared to CD36 expression in adipose tissue [28], skeletal [8,9,134,135,136,137] and cardiac [10,31] muscle expression was not upregulated, and change in total CD36 cell level was not observed in different insulin resistance animal models, but CD36 was permanently relocated to the sarcolemma from the intracellular storage compartment in response to FA oversupply. It is responsible for the increased rate of FA transport in such a way that it is no longer tuned to the metabolic needs of the myocytes. The recognized physiological factors regulating CD36 translocations in muscles are insulin with activation of PI3K pathway (FA uptake and esterification) and muscle contraction with the activation of AMPK pathway (FA uptake and oxidation) [108,138]. Both pathways converge at the level of the Rab GTPase-activating protein, AS160, via an inactivation of phosphorylation, which predisposes the cell to CD36 translocation. Insulin and muscle contraction are also the factors stimulating glucose transporter type 4 (GLUT-4) translocation [108]. However, according to Aguer et al. [137] other factors are probably responsible for permanent membrane CD36 relocation in insulin resistance. The mechanism of translocation was evaluated by Liu et al. [139]. They indicated that translocation as a result of palmitate overexposure causes the endosome to undergo alkalinization by the inhibition of proton pumping activity of vacuolar-type H^+^-ATPase (v-ATPase), and CD36 translocation is initiated. The inhibition of v-ATPase involves disassembly of subcomplex V_1_ from V_0_. Procedures which cause v-ATPase re-assembly, endosomal acidification, or CD36 retention lead to the reduction of myocellular lipid deposition and the preservation of insulin-stimulated GLUT-4 translocation, glucose uptake, and contractile function [26]. In contrast, Zhu et al. [140] studied a molecular pathway initiating sarcolemmal translocation of CD36 upon FA oversupply. They determined that CD36 translocation is induced via dual modulation of PKCζ and TBC1D1, but the association of these proteins; activation with endosomal alkalinization was not the subject of the study.

Lipid overexposure that changes the pH of endosomes not only contributes to the traffic of CD36 to sarcolemma, but also to GLUT-4 from endosomes to non-endosomal storage, in which it becomes trapped. The translocation of both is a vesicle-mediated process requiring specific vesicle-associated membrane proteins (VAMPs) [141]. The manipulation of selective VAMP expression in cardiomyocytes is a potential way to prevent the development of insulin resistance [142]. The relocation of CD36 precedes the onset of muscle insulin resistance with GLUT-4 retained intracellularly and decreases the incorporation of glucose into glycogen, the development of which is a long-term process, which requires the participation of many mechanisms. The role of CD36 in disrupting glucose metabolism was suggested in *CD36^−/−^* mice, in which the reduction in intramyocellular lipids was associated with an increased insulin-stimulated glucose transport, and a high-fat diet did not impair glucose tolerance [92].

Increased FA uptake occurs along with increased accumulation of TAGs, DAGs, and CERs in skeletal [92,135,136,137] and cardiac [10,31] muscles. In the case of a high-fat diet in rodents, the accumulation of intramyocellular lipids is very fast (within 2–3 days) [92]. As already described for the liver, elevated storage of intracellular TAGs is a marker of disordered FA metabolism, and DAGs and CERs are bioactive lipid intermediates inducing insulin resistance [91,123] by interfering with insulin signaling. That is associated with an impairment of the translocation of GLUT4 from endosomes to the sarcolemma, which results in decreased glucose transport into muscles and decreased incorporation of glucose into glycogen, and thus impairs contractile function [93,108]. The action of CERs is based on the stimulation of signal proteins which prevent the activation of protein kinase B (PKB/Akt) in response to insulin, such as PKCζ/λ or protein phosphatase 2 (PP2A) [123]. However, the role of CERs as a causative factor of insulin resistance is currently the subject of debate. The researchers’ positions were presented in *The Journal of Physiology*, in which arguments for and against the role of CERs in insulin resistance were presented [143,144].

The contribution of CD36 to the modulation of mitochondrial function in insulin resistance is not clear. Furthermore, the changes in FA oxidation induced by insulin resistance significantly differs between skeletal and cardiac muscles. There is a body of evidence of the defects in the FA mitochondrial oxidation and oxidative capacity of skeletal muscle in obesity, insulin resistance, and T2DM [145,146]. In contrast, there are reports that it is not mitochondrial dysfunction, but a reduction in the number of mitochondria that causes a reduction in β-oxidation. There is also a report claiming that β-oxidation is not impaired in insulin resistance subjects [132]. Regardless of this, it is clear that the reduction of FA oxidation deepens intramyocellular lipid accumulation. It was found that increased uptake of FA via CD36 into insulin resistance skeletal muscle was not associated with the changes in the rates of FA oxidation [92,135,136]. The situation in the myocardium is different, wherein FA oxidation is significantly increased at an early stage of insulin resistance. The activation of PPARα by FA oversupply leads to the upregulation of proteins involved in FA utilization and pyruvate dehydrogenase kinase-4 [31]. Intensified FA oxidation and decreased glucose oxidation are associated with a decreased cardiac efficiency, partially by the increased oxygen costs associated with the development of ROS-mediated mitochondrial uncoupling [31,147,148]. Chronic lipid overload of the heart in DM will ultimately lead to an inflammation and remodeling, and then diabetic cardiomyopathy, which is discussed in detail in the section about complications of DM. The contribution of CD36 to the pathogenesis of insulin resistance in muscles and the heart is summarized in Figure 2.

It is worth mentioning here that dietary FAs are capable of modulating the deleterious effects of insulin resistance by alterations to the functionalities of membrane proteins involved in insulin activity—among others, CD36—and their effects on the metabolism of glucose and FAs [149].

### 5.2. Pancreatic β-Cell Dysfunction and Damage

While changes in nutrition and lifestyle leading to weight loss allow restoration of tissue sensitivity to insulin, persistent hyperglycemic condition and high FFA level may culminate in an irreversible pancreatic β-cell failure, because they cause impaired function and damage to β-cells. These events accelerate the development of fasting hyperglycemia and T2DM [13,150].

CD36 is expressed in insulin-producing cells, such as MIN-6 cells, INS-cells, and human β-cells, where it mediates the regulation of insulin secretion [12,151,152]. Plasma FAs are the factor stimulating pancreatic β-cells to secrete insulin, but they require the presence of glucose. An increase in FFA level in insulin resistance is partially responsible for the typical high basal insulin response to glucose [107]. However, chronic exposure of β-cells to high levels of glucose and FFAs may lead to decreasing glucose-stimulated insulin secretion and development of T2DM by the induction of β-cell dysfunction and damage. Reduced β-cell function is an early and central event in the pathogenesis of T2DM and is already observed in prediabetes. In contrast, cell damage leading to a reduction of β-cell mass over time plays a secondary role [13,150,153]. CD36 contributes to both dysfunction and damage to β cells.

A high glucose condition stimulates CD36 expression in β-cells [154]. Overexpression of CD36 is associated with dysfunction of insulin secretion through increasing the FA influx. A high intracellular FA level promotes oxidative and ER stresses, and decreased insulin mRNA expression secretion. Inhibition of CD36 and reduction of CD36 expression can reverse these adverse effects induced by high glucose and improve glucose-stimulated insulin secretion [151,155,156]. The cellular mechanism of inhibition of glucose-stimulated insulin secretion by long-term exposure to the FFAs has been studied [157,158]. The authors concluded that FAs reduce insulin secretion at the exocytosis level [157] through dissociation of calcium channels from the secretory granules [158]. In contrast, Nagao et al. [12] found that CD36 overexpression present in the islets of obese patients with T2DM, but not those without T2DM, contributes to defective insulin exocytosis with the reduction of exocytotic protein levels and subsequent impairment of granule docking, which is associated with the reduction of the first-phase insulin secretion in T2DM. The molecular mechanism initiated by CD36 is the suppression of the insulin-signaling PI3K/AKT pathway and its downstream transcription factors. The observation that only obese human individuals that develop T2DM have higher CD36 expression than those who do not develop T2DM has also been reported in adipose tissue [28]. This provides evidence for the importance of predisposition for T2DM in obese individuals that may mediate both insulin resistance and impaired insulin secretion dependent on CD36. Furthermore, a critical effector in β-cell dysfunction and failure is the accumulation of CERs. An increased FA influx causes the accumulation of CERs (de novo synthesis) not only in hepatocytes and myocytes but also in pancreatic β-cells. Karunakaran et al. [159] have shown that incubation of insulin-producing cells with C2-CER causes an increase in CD36 protein level, reductions of insulin and PDX1 mRNA expression, and a reduction in cell apoptosis. PDX1 is an important regulator of insulin gene expression and β-cell development, differentiation, and survival. In a subsequent study, they found that CD36 activates of Src tyrosine kinase which mediates redoxosome (Vav2-Rac1- NADPH oxidase (NOX)) formation in response to C2-CER [11]. The activated complex initiates two different signaling pathways promoting pancreatic β-cell dysfunction and damage. The first includes the activation of NF-κB, and then the induction of thioredoxin-interacting protein (TXNIP) expression, an endogenous inhibitor of thioredoxin in β-cells. TXNIP promotes a range of effects, including modulation of the gene expression by altering the cellular redox state [159]. The second involves sequential activation of JNK, phosphorylation of serine36 of p66Shc, and hyperoxidation of peroxiredoxin-3 [11]. In both cases, the triggered mitochondrial stress initiates the reduction of glucose-stimulated insulin secretion and the intrinsic mitochondrial death pathway in β-cells. Another mechanism of FA-induced β-cell dysfunction is inflammation. Saturated FAs induce NF-κB activation and ER stress. This process may lead to a local chemokine release and islet inflammation [160]. A similar CD36-dependent mechanism was observed in adipose tissue in which the infiltration of macrophages was stimulated by the inflammatory response of adipocytes [6]. Intra-islet accumulation of macrophages with chronic low-grade inflammation may result in β-cell hyperplasia, impaired insulin secretion, and finally, β-cell failure [161,162,163]. The role of CD36 in this process has not yet been studied, but referring to previous reports, this is a promising research goal. Detailed information on the role of oxidative stress in pathomechanisms of β-cell dysfunction induced by hyperglycemia and elevated FFAs was described by Newsholme et al. [164].

Lipid and glucose toxicity also underlie the mechanism of β-cell damage, and this phenomenon has been termed glucolipotoxicity. They have already been partially described above. In a high glucose state, the increased influx of FA into β-cells leads to the production of large amounts of reactive oxygen species (ROS) by the Rac1-NOX complex, followed by mitochondrial dysfunction and β-cell apoptosis [154]. CD36 suppression of insulin-producing cells attenuated apoptosis induced by a high glucose condition [156], FAs [165], and CERs [11,159]. Glucolipotoxicity conditions may also regulate CD36 functional activity, post-translationally. Khan et al. [166] indicated that the initiation of functional activation of CD36 requires lysine deacetylation, but specific lysine deacetylases have not been yet identified. The consequences of this modification are lipid accumulation and caspase 3 activation in β-cell. Sulfosuccinimidyl oleate (SSO), an irreversible inhibitor of CD36, prevents these deleterious effects induced by glucolipotoxicity.

In summary, CD36 has become a promising target molecule for preventing glucolipotoxicity in β-cells. Hitherto, the effects of several drugs commonly used in diabetics have been assessed. Ezetimibe [151] and metformin [156] may prevent glucotoxicity through a decrease of CD36 expression and FA influx, due to the reversed suppression of insulin secretion in INS-1 cells and primary rat islet cells. Fenofibrate limits β-cell dysfunction and apoptosis caused by lipotoxicity through the inhibition of the NF-κB/MIF inflammatory pathway. The improvement of glucose-stimulated insulin secretion and β-cell mass was also observed. CD36′s contribution was not evaluated in this study, but it seems very likely [167].

## 6. Diabetic Complications

One of the most important clinical manifestations of DM is the development of chronic tissue complications. It particularly concerns such organs as the kidneys, eyes, peripheral nerves, heart, and blood vessels. It is important to remember that DM dysregulates the metabolism in a tissue-specific manner. The factors that underlie diabetic complications can be divided into two groups, including the effectors associated with hyperglycemia or alterations in systemic and local lipid metabolism [4].

### 6.1. Hyperglycemia

Short-term hyperglycemia is not associated with serious damage. However, persistent hyperglycemia is a factor initiating damage to sensitive tissues proportional to the severity of glycemic abnormalities. The results of persistent hyperglycemia are: (1) an uncontrolled formation of ROS [168] and (2) non-enzymatic glycation of structural and functional proteins with the formation of AGEs, which accumulate intra cellularly and extracellularly [169].

Excessive ROS production with an inefficient antioxidant system can induce oxidative stress, promoting the oxidation of proteins, lipids. and nucleic acids. We would like to pay special attention to advanced oxidation protein products (AOPPs), oxLDL, and oxidized high-density lipoprotein (oxHDL). AOPPs are dityrosine cross-linked and carbonyl-containing proteins produced in a reaction between chlorinated oxidants (chloramines and hypochlorous acid) with plasma proteins, especially albumin. Plasma AOPPs levels are increased in DM and they are associated with the pathogenesis of microvascular or macrovascular complications of DM [168,170]. Their mechanisms of action are less known than AGEs, but so far it has been established that AOPPs interact with a similar set of receptors, e.g., RAGE [171] and CD36 [172]. The pathogenic role of AOPPs was confirmed in chronic kidney disease, atherosclerosis, and cardiovascular events via the redox-dependent pathways [173]. Typical of DM is also an increased formation of lipoprotein oxidation products, for instance, oxLDL and oxHDL [174]. Importantly, HDLs undergoing oxidative modification lose their protective properties, and LDL acquires proinflammatory, proapoptotic, and proatherogenic properties [174,175]. The associations of both oxLDL and oxHDL with atherosclerosis [68,174], nephropathy [175,176,177], and retinopathy [178] were confirmed.

AGEs are formed in a non-enzymatic reaction, known as the Maillard reaction, between the carbonyl groups of reducing sugars and the free amino groups of proteins. Pathogenicity of AGEs is related to their interactions with a heterogonous group of plasma membrane receptors, including RAGE (receptors for AGEs), lactoferrin, class A scavenger receptors type I and II (SR-A), CD36, and others, which alter intracellular signaling, gene expression, and the release of pro-inflammatory molecules and free radicals [169]. CD36 binds AGEs using a domain, which, to some extent, overlaps with the binding domain for oxLDL [60]. A large group of receptors for AGEs are expressed in certain sensitive cells in response to DM, such that AGEs play an important role in the pathogenesis of diabetic complications such as nephropathy, retinopathy, neuropathy, and cardiomyopathy [169].

### 6.2. Alterations in Lipid Metabolism

Dyslipidemia is a common feature in patients with DM, especially T2DM, which includes both quantitative and qualitative alterations in plasma lipoproteins. Quantitative lipid abnormalities may involve increases of TAGs, non-HDL cholesterol, and small dense LDL, and a decrease of HDL cholesterol, depending on the types of DM and glycemic control respectively [179,180,181]. Another disturbance is an increased plasma level of FFAs, which are harmful to various tissues due to their lipotoxicity [182]. Qualitative alterations rely on lipoprotein modifications, such as the oxidation or glycosylation mentioned above. The development of diabetic nephropathy (DN) and its progression intensify the disturbances of lipoproteins metabolism [179]. An abnormal profile of plasma lipids is a risk factor for progressive renal disease [183], premature atherosclerosis, and cardiovascular disease—the main cause of morbidity and mortality in patients with T1DM and T2DM [180,181].

## 7. The Role of CD36 in Diabetic Complications

CD36 is a mediator of multiple pathways that perform important roles in the pathogenesis and progression of diabetic complications.

### 7.1. Nephropathy

Nephropathy is one of the most common diabetic microvascular complications and the major cause of the end-stage renal disease (ESRD) in developed countries [184]. DN is estimated to affect one-third of humans with DM and is associated with prominent cardiovascular morbidity and mortality [185]. DN is defined as the progressive loss of kidney function resulting from the effects of both T1DM and T2DM on the kidney [186]. This is due to the fact that the kidneys are sensitive to hemodynamic (systemic and glomerular hypertension) and metabolic (hyperglycemia and hyperlipidemia) disruptions caused by DM [187,188]. The overt nephropathy develops within 10–15 years after the onset of DM [186,188].

DN is characterized by structural and functional changes in glomeruli, tubules, vasculature, and interstitium. Structural abnormalities of the glomeruli include glomerular hypertrophy, thickening of the basement membrane, expansion of mesangial extracellular matrix, and nodular glomerulosclerosis. At an early stage. tubular hypertrophy is present but the gradual loss of kidney function at later stages of DN is associated with interstitial fibrosis and tubular epithelial degeneration (tubular atrophy), hallmarks of degeneration to ESRD, along with arteriolar hyalinosis. Advanced stages are accompanied by infiltration of macrophages and T-lymphocytes [188]. DN is clinically manifested by hyperfiltration and albuminuria (urinary albumin to creatinine ratio ≥ 30 mg/g) in the early phase, which are then followed by an ultimate decrease in the estimated glomerular filtration rate (eGFR < 60 mL/min/1.73 m2) and the progression of chronic kidney disease to ESRD [189,190].

CD36 is expressed in many kidney cells—podocytes, mesangial cells, proximal and distal tubular epithelium, microvascular endothelial cells, and interstitial macrophages [55]. This receptor plays a significant role in the progression of DN by (1) the initiation of renal lipid deposition [16,191] and (2) hyperglycemia or the interaction with products formed during chronic hyperglycemia, such as oxidized lipoproteins, AGEs, or AOPPs [172,176,183,192]. These cellular events promote inflammation, and oxidative and ER stress, resulting in kidney damage. Significant deposition of lipids in the kidney tissue was observed in glomerular and tubular cells, but especially in podocytes, of patients with DM. The amount of deposited lipids varies depending on the stage of DN and it is reduced in advanced fibrotic kidneys. TAG accumulation is promoted by increased uptake of FAs (increased expression of CD36) and reduced FA β-oxidation (decreased expression of acyl-CoA oxidase and carnitine palmitoyltransferase I) by a downregulation of PPARα and PPARδ. Cholesterol accumulation is associated with the upregulation of lipoprotein receptors, such as oxidized low-density lipoprotein receptor 1 (LOX-1/OLR-1), low-density lipoprotein receptor (LDLR), SR-A1 (scavenger receptor for acetylated LDL), and CD36, and a downregulation of the cholesterol efflux genes, ATP-binding cassette transporters (ABCA1, ABCG1). These alterations of the metabolism of FAs and cholesterol genes were correlated with the reduction of eGFR [191]. In addition to lipid deposition, reduced FA oxidation in tubule epithelial cells causes ATP depletion, cell death, and dedifferentiation, and favors fibrosis [193]. The pathogenic mechanisms, mediated by an abnormal cellular lipid metabolism, are based on inflammatory activation and profibrotic responses.

**Figure 3:**
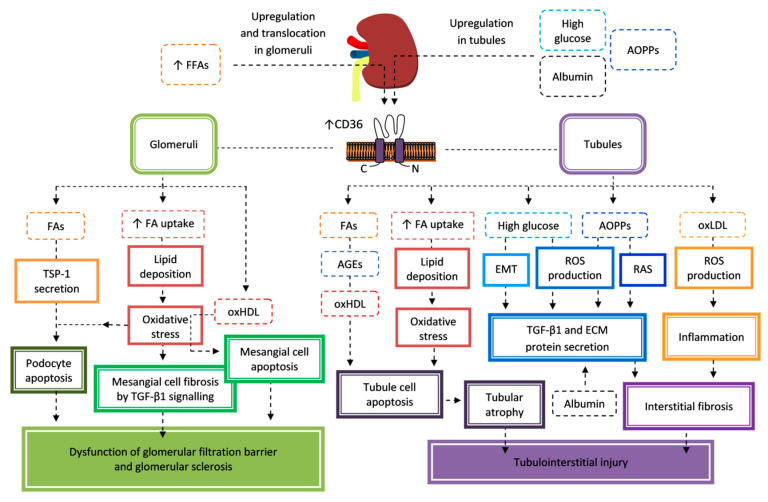
The contribution of CD36 to the pathogenesis of diabetic nephropathy (DN). CD36 is involved in both glomerular and tubular damage in diabetes mellitus (DM). Increased fatty acid (FA) uptake with lipid deposition is the main cause of glomeruli damage by induction of podocyte apoptosis, but also causes the initiation of glomeruli sclerosis by transforming growth factor β1 (TGF-β1) signaling. Moreover, oxidized high-density lipoprotein (oxHDL) induces mesangial cell apoptosis. These effects promote the dysfunction of the glomerular filtration barrier. Many more factors are involved in tubule cell damage. As with podocytes, apoptosis is induced by lipotoxicity in tubule cells. Furthermore, hyperglycemia, the products of lipid and protein oxidation (oxHDL, oxidized low-density lipoprotein (LDL), advanced oxidation protein products (AOPPs), and advanced glycation end products (AGEs)) mediate apoptosis of tubular cells or fibrosis of the interstitium, resulting in tubulointerstitial injury. Interstitial fibrosis is initiated by the epithelial-to-mesenchymal transition (EMT) in response to hyperglycemia, but also the secretion of TGF-β1 and extracellular matrix (ECM) protein mediated by reactive oxygen species (ROS), or activation of the renin-angiotensin system (RAS) in response to AOPPs and oxLDL. These various CD36-dependent mechanisms ultimately lead to a progressive loss of kidney function in DM.

As already mentioned, DM is often accompanied by a high level of saturated FFAs which is a lipotoxic factor. This also applies to the kidney tissues in which FFAs promote DN progression by increasing CD36 expression. Palmitic acid induces CD36 expression in podocytes [14] and mesangial cells [16] of glomeruli and stimulates the translocation of CD36 from the cytoplasm to the plasma membrane, leading to increased lipid uptake and deposition [14,194]. Lipid accumulation initiates oxidative stress responsible for the induction of podocyte apoptosis [14,182,195] and mesangial cell fibrosis [16]. Mitochondrial and cytoplasmatic ROS production causes dysfunction of mitochondria and ER with Ca^2+^ depletion [182]. Induced ER stress and the intrinsic mitochondrial apoptotic pathway (but not the death receptor-mediated pathway) have been reported to be involved in apoptosis of podocytes activated by palmitic acid [182,195]. ROS production induced in mesangial cells by palmitic acid is accompanied by an increase of transforming growth factor β1 (TGF-β1); and signaling pathway proteins, including p-Smad2/3, fibronectin, collagen α-1 (IV) chain (Col4 A1), NOX4, and p22phox, which promote fibrosis of glomeruli [16]. FFAs also induce the expression of TSP-1 in podocytes. Secreted TSP-1 interacts with CD36 and mediates FFA-induced apoptosis of podocytes without the activation of the TGF-β pathway, which is one of the pathways responsible for the induction of podocyte apoptosis [196]. Moreover, oxLDL [176] and oxHDL [175] are known for pro-inflammatory and cytotoxic actions on glomeruli and tubules in DM. OxLDL is taken up by podocytes, mainly by C-X-C motif chemokine ligand 16 (CXCL16), but not by CD36. Therefore, CD36 is of little importance regarding the harmful effects of oxLDL in glomeruli [176]. However, oxHDL enhances ROS production and inflammation in mesangial cells mediated by CD36 and LDLR, which activate p38 mitogen-activated protein kinase (MAPK), ERK/MAPK, and NF-κB pathways. These alterations predispose to dysfunction and apoptosis of mesangial cells [177]. Thus, CD36 mediates lipid deposition in glomeruli cells, which results in glomerular sclerosis and the loss of podocytes with the failure of an essential component of the glomerular filtration barrier, proteinuria, and the progressive loss of kidney function in DM patients [14] (Figure 3).

CD36 is also involved in a number of pathogenic mechanisms resulting in tubulointerstitial injury in the kidney, including tubular atrophy and interstitial fibrosis. Several factors initiate tubular epithelium atrophy by the induction of apoptosis in the CD36-dependent mechanisms. High ambient glucose induces CD36 expression in human proximal tubular cell line HK-2 [15,192,197] by upregulating the Akt-PPARγ signaling pathway, leading to lipid deposition [15]. Increased CD36 expression is essential for the activation of a proapoptotic signaling pathway [192]. By promoting intracellular lipid accumulation, CD36 can increase ROS production, trigger ER stress, and induce inflammatory responses, which are associated with a decrease in renal tubular cells’ viability [15]. Other factors initiating apoptosis of proximal tubular epithelial cells are glycated albumins and palmitate. The activated signaling pathway includes the activation of Src kinase, proapoptotic p38 MAPK, and caspase 3 [192], as in the cases of the studies on macrophages [198] and vascular endothelial cells [199]. Elevated levels of AGEs [200,201] and FAs [202] were found in the urine of patients with DN, suggesting that their accumulation in the tubular cells contributes to damage of the renal tubules. Moreover, CD36 contributes to interstitial fibrosis mainly by the activation of the pathways that result in TGF-β and ECM protein secretion. CD36, in response to high glucose levels, mediates epithelial-to-mesenchymal transition (EMT) in tubular epithelial cells [203]. EMT involves the acquisition of mesenchymal properties by renal epithelial cells. The renal epithelial cells become myofibroblasts that synthesize and secrete ECM proteins (collagen, laminin, and fibronectin) deposited in excess in the renal tubulointerstitium, contributing to fibrosis [204]. The inhibition of CD36 reduces hyperglycemia-induced ROS production, activation of the ERK1/2 and Smad2 signaling pathway, and expression of prosclerotic cytokine TGF-β1 and ECM protein fibronectin [203]. Albumin filtered through damaged glomeruli may also induce proximal tubules fibrosis by an upregulation of CD36 with a concomitant increase of TGF-β1 and fibronectin secretion [205]. Other effectors of tubulointerstitial injury are AOPPs. CD36 is involved in the uptake of AOPPs, which is a factor upregulating CD36 and stimulating the production of ROS and TGF-β1 in tubular cells [172]. AOPPs accumulate in tubular cells and activate the intrarenal renin–angiotensin system (RAS) in a CD36-dependent mechanism. AOPP-albumin interaction with CD36 initiates redox-sensitive signaling consisting of activation of PKCα, NOX, and NF-κB/activator protein 1 (AP-1). The activation of RAS could stimulate inflammation, apoptosis, fibroblast activation, or ECM overproduction favoring the progression of renal fibrosis [206]. AOPPs also promote lipid accumulation associated with lipotoxicity and fibrosis by an upregulation of CD36 and the activation of the CD36-dependent pathways, including Wnt/β-catenin activation [17]. Unlike in podocytes, CD36 is important in the uptake of oxLDL in tubular cells [176]. High levels of circulating oxLDL and cardiotonic steroid associated with hyperlipidemia induce oxidative stress and inflammation in the kidney by an interaction with CD36 and Na/K-ATPase, in both proximal tubule cells and their associated macrophages. The authors suggest that these transmembrane proteins act synergistically because they share a common signaling step, including the activation of Src kinases and ROS generation, resulting in proinflammatory cytokine release by both proximal tubule cells and macrophages, thereby enhancing an inflammatory paracrine loop between these adjacent cells that may contribute to tubulointerstitial fibrosis [183]. Like oxLDL, oxHDL stimulates ROS production and upregulation of proinflammatory factors (TNF-α, MCP-1, and RANTES) in proximal tubule cells by the CD36-dependent mechanism, including the activation of the Src, MAPK, and NF-κB pathways. It is associated with a reduction of cell migration and an increase of apoptosis [175]. In summary, CD36 mediates apoptosis of tubular cells induced by hyperglycemia, glycated albumin, FAs, or oxHDL, while hyperglycemia, albumin, AOPPs, or oxLDL promote interstitial fibrosis by EMT, the secretion of ECM proteins, and TGF-β1 and inflammation. Ultimately, these mechanisms lead to tubular atrophy and interstitial fibrosis, and thus kidney dysfunction (Figure 3).

These studies indicate that CD36 can be a potential therapeutic target for DN, both at the level of glomeruli and tubules, and interstitium. Hitherto, the focus has been on compounds that prevent oxidative stress, particularly important in the development and progression of DN, caused by CD36. SS31 is a mitochondria-targeted antioxidant peptide which protects against renal pathological damage induced by hyperglycemia. The renoprotective effect was mediated by suppression of the CD36-dependent lipid accumulation, NF-κB signaling, and oxidative stress resulting from NOX-mediated ROS production and downregulation of the antioxidant enzymes superoxide dismutase and catalase. The administration of SS31 in db/db mice improves kidney function by mitigating glomerular hypertrophy, tubular injury, and proteinuria [197]. Astragaloside IV, glycoside from the astragalus plant known for its antioxidant and anti-inflammatory properties, inhibited palmitate-induced oxidative stress and fibrosis in mesangial cells by the reduction of CD36 expression, thereby decreasing FFAs uptake and lipid deposition [16].

In reference to the role of CD36 in DN pathogenesis, the utility of plasma and urine sCD36 as a DN biomarker was evaluated. It was indicated that plasma and urine sCD36 levels are increased in patients with DM and correlate with the severity of DN rated by kidney markers, such as albuminuria, urea, creatinine and eGFR. Therefore, sCD36 has a prognostic value and enables monitoring of the course of DN [207].

### 7.2. Retinopathy

Retinopathy is another microavascular complication of DM which affects one in three people with DM. It is estimated that diabetic retinopathy (DR) will develop in up to 90% of T1DM patients and in 50–60% of patients with T2DM [208]. DR is defined as a progressive retinal damage due to DM. The risk of the development and progression of DR is increased along with the duration of DM, hyperglycemia, hypertension, and dyslipidemia. An early stage of DR is non-proliferative retinopathy (NPDR) which progress into an advanced stage called proliferative diabetic retinopathy (PDR). Characteristic features of NPDR are microaneurysms, intraretinal hemorrhages or venous beading, and PDR neovascularization, vitreous hemorrhage, or tractional retinal detachment. DR may be accompanied by diabetic macular edema (DME) with fluid accumulation within the central neural retina, which is independent of DR progression and requires a separate evaluation [209,210]. DR is the main reason for blindness and moderate-to-severe vision loss in working-age adults, but early diagnosis and proper management of patients can prevent more than 90% of cases of visual loss [208,210].

DM-induced metabolic abnormalities of the retina environment cause oxidative stress and inflammation which initiate damage and dysfunction of the retina. DR is a result of neurodegeneration and vasculopathy leading to retina ischemia, permeability and neovascularisation, and macular edema [210,211]. Recently, neuroretinal degeneration has been recognized as an early event in DR, which precedes symptomatic vasculopathy. Underlying pathological events in retinal neurodegeneration are: neural apoptosis, reactive gliosis, glutamate excitotoxicity, reduction in neuroprotective factors, and deterioration of the neurovascular coupling [211]. Neuronal damage is accompanied by increased expression and release of vascular endothelial growth factor (VEGF), which on the one hand is neuroprotective and on the other hand is a factor initiating neovascularization and PDR. In the classical approach, the main pathological roles are played by microvascular alterations, including the loss of pericytes and endothelial cells and basement membrane thickening, and the changes in the rheological properties of the blood, which together lead to capillary occlusion and degeneration [209,212]. DM not only causes damage to the retinal endothelial cells and inner blood-retinal barrier, but also a dysfunction of retinal pigment epithelium (RPE) layer expressed as disruption of transport by an outer blood-retina barrier (BRB) [213] and imbalance of secretions of cytokines, chemokines, and growth factors [214].

The CD36 scavenger receptor features prominently in ocular homeostasis and pathology [215]. It is expressed in retina cells, such as pericytes [19], endothelial cells [216], RPE [217,218], and infiltrated mononuclear phagocytes [20]. CD36 participates in the pathogenesis of DR through a number of effects induced in retinal cells in response to hyperglycemia, FFAs, and modified lipoproteins.

Hyperglycemia is a well-known factor that damages microvascular endothelial cells. High glucose upregulates CD36 expression and oxLDL uptake in endothelial cells [32]. Moreover, CD36 interacts with endothelial nitric oxide synthase (eNOS) and mediates oxidative cell damage. OxLDL potentiates microvascular injury [219]. In human retinal microvascular endothelial cells, palmitate interacts with lipopolysaccharide, both of which are increased in T2DM, to upregulate IL-6 expression via CD36 [18].

A significant event in the diabetic retina is the dysfunction of BRB, which causes lipoprotein leakage into retina. Human plasma oxLDL represents only a small fraction of total LDL, but most of it is modified into glycated oxLDL (glc-oxLDL) [220]. In an atheromatous plaque, the oxLDL concentration is much higher than in plasma and is associated with plaque instability [221]. It is similar in the diabetic retina [222]. However, there is a significant difference between the accumulation of modified lipoproteins in the arteries and the retina. The accumulation and modification of LDL in the arteries lasts throughout life. In contrast, accumulation in the retina occurs only when it comes to BRB abnormalities, as in DM. LDL leakage is prevented in individuals with normal BRB retinas [223]. Intra-retinal lipoproteins undergo extensive modification and are gradually accumulated, leading to damage. Wu et al. [224] demonstrated that oxLDL was present in diabetic human retinas, even before the development of clinical retinopathy, but were absent in nondiabetic retinas. The presence and increase of oxLDL and glc-oxLDL in a DM cohort predicted DR progression [225]. Modified LDLs have a cytotoxic effect on retinal cells consisting of increased oxidative stress and decreased viability of retinal vascular cells (endothelial cells, pericytes) [19,226,227,228] and RPE cells [218,223]. CD36 is one of the receptors for oxidatively modified lipoproteins and promotes their uptake [229].

The loss of vascular integrity is intensified by the modified lipoproteins. Pericytes are critical for maintaining the correct shape and permeability of microvessels and inner BRB. Glc-oxLDL induces autophagy or apoptosis of retinal pericytes depending on the severity of experienced cellular stress [227,228]. The autophagy in retinal cells helps maintain the retina’s structure and function, and its upregulation in retinal rods is an early feature of DR in the mouse model of DM [230]. When glc-oxLDL causes relatively mild stress in pericytes, such as in early DR, autophagy improves cell survival. However, when stress is severe, such as in advanced DR, autophagy loses its cytoprotective effect and promotes the disruption of cellular homeostasis and apoptosis [228]. CD36 is a promising candidate for mediating this mechanism, but this requires confirmation. It is also well-known that the modification of LDL may render it immunogenic [231]. Some data support the hypothesis about immunogenic oxLDL cytotoxicity in the retinal cells. Scavenger receptor CD36 for oxLDL and other multi-molecular complexes of molecules, along with the CD64 receptor, which has a high affinity to Fcγ of IgG, were detectable in pericytes. A study on cell cultures demonstrated that the oxLDL immune complex has greater cytotoxicity than oxLDL alone and it leads to pericyte apoptosis. Moreover, an increase of inflammatory cytokines and a reduction of a key anti-angiogenic factor pigment epithelium-derived factor’s (PEDF) secretion are induced. Pericyte loss is associated with the formation of microaneurysms and intraretinal hemorrhages. Immune complex depletion in the retina favors the infiltration and activation of macrophages and angiogenesis, resulting in the progression to PDR [19].

Harmful effects of modified LDL in the retina extend beyond vascular cells and include the outer BRB formed by RPE cells. The RPE cells are able to uptake oxLDL via CD36 and are key regulators of lipid metabolism in the retina [218]. The effect of oxLDL on RPE cells is associated with the development of age-related macular degeneration (AMD). OxLDL has been shown [232] to accumulate preferentially in the macula of patients with AMD, so the pathogenesis of AMD shares many features with the development of atherosclerosis, including the association with oxidized lipid accumulation and inflammation [233,234]. The oxLDL uptake by RPE in vitro and in vivo is CD36-dependent and prevents age-dependent sub-retinal laminar deposits of oxidized lipids [217]. RPE cells clear oxLDL until they are overloaded with oxLDL. Then oxLDL increases the expression of CD36 in human RPE cells, which mediates cell death by NLRP3 inflammasome activation [218]. While, glc-oxLDL induces oxidative stress, ER stress, and autophagy in RPE cells, and eventually cell apoptosis by activation of the proapoptotic transcription factor CCAAT/enhancer-binding protein homologous protein (CHOP), HDL prevents this action, but its modification reduces its antioxidative action. The contribution of CD36 to this mechanism is not confirmed [223]. However, it is clear that modified LDLs may deprive many functions of RPE, among others, the protection of the macula against the adverse effects of deposited modified LDLs and maintaining integrity of the outer BRB. Given the pathogenesis of DR, the involvement of CD36 in RPE dysfunction in DR is an important issue that requires further study.

The progressive increases in vascular permeability and cytokine release in DR promote retinal infiltration by mononuclear phagocytes and the inflammatory response [235]. Mononuclear phagocytes (macrophages and microglia) accumulate in subretinal space, where they mediate inflammasome-dependent injury of photoreceptors and RPE. CD36 and the TLR2/6 heterodimer are co-expressed in the cell membrane of mononuclear phagocytes and their interaction promotes the degeneration of the retina by chronic release of proinflammatory cytokines. MPE-001, an azapeptide ligand of CD36, is cytoprotective as it modulates the inflammatory profile of mononuclear phagocytes, which protects photoreceptors and prevents against vision loss [20]. Similar protective effects were observed in CD36-deficient mice [236].

The persistent microvascular non-perfusion results in ischemia in the diabetic retina, which is associated with the development of PDR and DME. Ischemia may induce hypoxia-related upregulation of cytokines, growth factors, such as VEGF, but also CD36 [237]. Unlike the mechanisms described above, CD36 also appears to be involved in the prevention of the progression to PDR by action as a negative feedback regulator of pathological angiogenesis. In RPE cells, the upregulation of CD36 expression under hypoxic stress via activation of hypoxia-inducible factor-1 (HIF-1) and PI3K pathways has recently been described [238]. HIF-1 binds to a conserved hypoxia response element (HRE) located in the *CD36* promoter, and CD36 transduces signals, leading to apoptosis-dependent inhibition of angiogenesis. Suppression of the PI3K pathway prevents hypoxia-induced CD36 promoter activity. Similar negative-feedback mechanisms during corneal neovascularization were reported in other studies [239,240], wherein CD36 activation suppressed angiogenesis by antagonizing the VEGF pathway. On the other hand, it was demonstrated [241] that the interaction of TSP-2 (glycoproteins with anti-angiogenic functions) with CD36 inhibits the migration and tube formation and induces apoptosis of microvascular endothelial cells. CD36 proteins are specifically localized in vascular endothelial cells. TSP-2 binding to the CD36 receptor mediates inhibition of neovascularization. The study [216] showed that the expression of CD36 in cell membranes from patients with active PDR was significantly higher than in membranes from patients with inactive disease. Therefore, physiological upregulation of TSP-2 may be not just the mechanism through which the ocular microenvironment protects itself against excessive blood vessel formation, but may also be a defense mechanism against excessive tissue inflammation.

**Figure 4:**
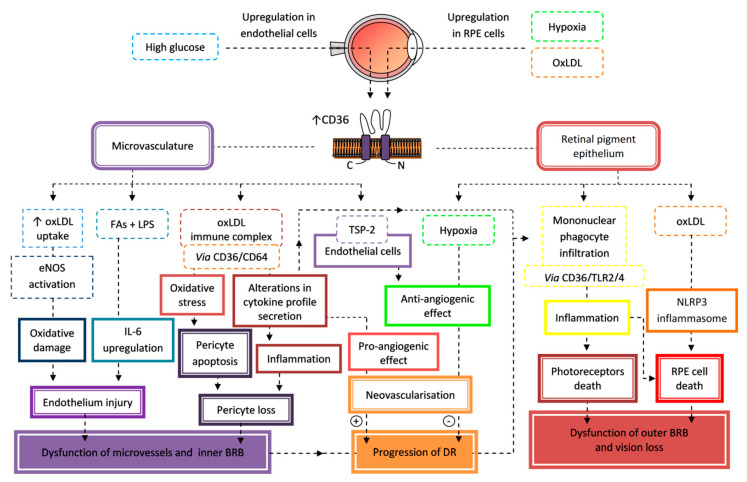
The contribution of CD36 to the pathogenesis of diabetic retinopathy (DR). CD36 is involved in both microvasculature and retinal pigment epithelium (RPE) damage of the retina. Microvascular cell damage is associated with oxidized low-density lipoprotein (oxLDL) uptake and endothelial nitric oxide synthase (eNOS) activation in endothelial cells or the response of pericytes to oxLDL immune complex. The OxLDL immune complex induces pericyte apoptosis, but also inflammation-promoting loss of pericytes and infiltration of the retina by mononuclear phagocytes. Induced dysfunction of the microvessels and inner blood-retina barrier (BRB) intensifies the infiltration of inflammatory cells. Retina inflammation mediated by mononuclear phagocytes or oxLDL also causes photoreceptors and RPE cell death. Moreover, the oxLDL immune complex stimulates the release of proangiogenic cytokine pigment epithelium-derived factor (PEDF), which may favor neovascularization and progression of DR. These events are associated with progressive retina injury and loss of vision. On the other hand, CD36 seems to prevent neovascularization in advanced retinopathy by the interaction of endothelial CD36 with anti-angiogenic thrombospondin (TSP-2) or the anti-angiogenic effect on RPE cells initiated by hypoxia.

Currently, the role of CD36 in DR cannot be clearly defined, but it seems to differ significantly depending on the stage of the disease and type of retina cells. CD36 mediates modified LDL-induced microvascular damage in the early stages of DN, but prevents neovascularization typical for progression to PDR. On the other hand, it promotes the proinflammatory profile of mononuclear phagocytes associated with damage to photoreceptors and RPE, and loss of vision (Figure 4).

### 7.3. Peripheral Neuropathy

The most prevalent complications of DM, affecting at least half of the patients, are neuropathies characterized by significant heterogeneity in terms of their clinical presentation, risk factors, and pathophysiology [242]. Among them, distal symmetric polyneuropathy, referred to as diabetic peripheral neuropathy (DPN) in this review, is the most common diabetic neuropathy syndrome (~75% of diabetic neuropathies) [243,244]. According to the Toronto Consensus Panel on Diabetic Neuropathy, DPN is defined as a symmetrical, length-dependent sensorimotor polyneuropathy attributable to metabolic and microvessel alterations as a result of chronic hyperglycemia exposure and cardiovascular risk covariates [245]. DPN occurs along with the loss of sensory function, beginning distally in the lower limbs, and in some cases causes neuropathic pain. It leads to disabilities due to foot ulcerations, a high risk of amputation, gait disturbance, and fall-related injuries [243,244].

As with other diabetic complications, the development of DPN has a multifactorial and complex pathogenesis. Peripheral nerve dysfunction is a result of disturbances caused by hyperglycemia and dyslipidemia within axons, Schwann cells, and microvessels. The events responsible for the initiation and progression of the DPN are the polyol pathway, oxidative and ER stress, inflammation, and mitochondrial dysfunction. Therefore, pathological features of DPN include axonal degeneration and necrosis, demyelination, Schwannopathy, and microangiopathy [19,22,243,245,246]. These pathological alterations have been described by Pande et al. [246]. They carried out their study on BKS db/db mice, a mouse model of T2DM, which allowed them to present the most important structural and metabolic changes occurring within the peripheral nerves in response to hyperglycemia and dyslipidemia. They include downregulation of genes associated with myelin formation and axonal regeneration; downregulation of neurotrophin signaling pathway genes; and modulation of anti and pro-apoptotic gene expression, predisposing cells toward apoptosis. They were associated with an impaired axonal transport, neurotrophic signaling, cell adhesion, and communication. The well-described metabolic abnormality was increased glucose metabolism, resulting in oxidative stress, inflammation, and axonal ischemia in the sciatic nerve. Another abnormality was the dysregulation of lipid metabolism, including the upregulation of genes of FA and glycerolipid metabolism, lipid transport, and PPAR signaling, which were likely related to axonal degeneration.

Among the diabetic complications we have described, the role of CD36 in the pathogenesis of DPN is the least well established. Gene expression profile studies in DPN identified several candidates associated with the lipid metabolism and inflammation potentially involved in the initiation and progression of this complication; among them is CD36 [21,247]. Increased CD36 expression in peripheral nerves was observed in BKS *db*/*db* mice [246] and individuals [27] with DPN. However, CD36 expression levels differ between patients with non-progressing and progressing DPN, in whom it was upregulated and downregulated, respectively [27]. This observation suggests the complex role of CD36 in the pathogenesis of DPN.

CD36 is implicated in the initiation of inflammation in peripheral nerves. The upregulations of CD36 and MAPK signaling pathway genes (*TNF-α*, *IL-1a* and *TGF-β1*) are closely associated with the nerves of BKS *db/db* mice and the authors suggest a CD36-mediated inflammatory response [246]. Moreover, it is suspected that CD36 also modulates energy homeostasis-related signaling pathways, such as AMPK and PPAR pathways, changing the glucose and lipid metabolism in diabetic peripheral nerves [247]. Dyslipidaemia was shown to predispose to the dysregulation of lipid metabolism in peripheral nerves correlated with upregulation of CD36 and diacylglycerol acyltransferase 2. Saturated FAs are incorporated into TAGs, which initiate nerve injury [22]. The influx of FAs is a known initiator of lipotoxicity. An exposure of Schwann cells to high levels of palmitate results in ER stress, ROS generation, and mitochondria depolarization, eventually leading to cell apoptosis. High glucose levels intensify this effect. This suggests that chronic exposure to hyperglycemia and FAs will result in Schwann cell dysfunction, demyelination, and axon atrophy [248]. The contribution of CD36 has not been assessed in this study, but considering the results of the aforementioned studies on other cells, it can be suspected that CD36 is a potential mediator for these pathways initiated by palmitate. In contrast, in progressive DPN, lipid metabolism of Schwann cells might be inhibited partially by the downregulation of CD36 and the reduction of lipid uptake [21]. Kim et al. [249] indicated that chronic exposure to high glucose reduces PPAR-γ binding to target genes in Schwann cells. This may be one of the causes of downregulation of CD36, whose expression is regulated by this transcription factor. However, is unclear whether this promotes or protects against demyelination [21]. On the one hand, demyelination is an early pathological feature in DPN, and a decrease in FA inflow will reduce the lipotoxic effect in Schwann cells, suggesting protective action of CD36 downregulation. On the other hand, there are suggestions that CD36 downregulation is harmful and may contribute to the limitation of fiber myelination. Inhibition of PPAR-γ function in response to hyperglycemia is associated with both reduced FA uptake and ineffective lipogenesis in Schwann cells, thereby reducing the supply of FAs necessary for effective myelination [250]. Moreover, PPAR-γ agonists improve DPN [249]. Given that demyelination is an important early feature of DPN, detailed research is required. Undoubtedly, CD36 is significant in the pathogenesis of DPN, but the mechanisms involved are very poorly understood, which gives researchers a wide field of action.

### 7.4. Cardiomyopathy

Diabetic cardiomyopathy (DCM) is a complication of DM with a silent and slow development, which ultimately leads to heart failure (HF) if left undiagnosed and untreated at an early stage. Diabetics have a higher risk of developing heart disease, including HF, compared to healthy individuals [251]. The prevalence of HF among diabetics ranges from 19% to 26% [252]. DCM is defined as a clinical condition of ventricular dysfunction that occurs regardless of other conventional cardiac risk factors, such as coronary atherosclerosis, hypertension, or valvular disease, in patients with DM. In early stages, DCM proceeds in a latent subclinical phase characterized by structural and functional abnormalities, including concentric hypertrophy and fibrosis of the left ventricle (LV), and cell signaling abnormalities. These structural and metabolic alterations of the myocardium are associated with subclinical diastolic dysfunction—the first hallmark of DCM. DCM evolves to HF with a preserved ejection fraction (EF) and the eventual systolic dysfunction accompanied by HF with reduced EF. The occurrence of HF significantly worsens the prognosis of patients with DM [252,253].

DCM initiating factors include hyperglycemia, systemic insulin resistance, and disturbed insulin signaling in cardiomyocytes, which promotes cardiac structural remodeling and diastolic and systolic dysfunction by stimulation of numerous metabolic abnormalities. They include oxidative and ER stress, inflammation, mitochondrial dysfunction, impaired calcium handling, extracellular matrix remodeling, but also activation of the renin-angiotensin-aldosterone system, cardiac autonomic neuropathy, and microvascular dysfunction [252].

CD36 is the main lipid transporter of cardiomyocytes’ contributing to the rate-limiting kinetic step in lipid utilization, and that appears to be necessary in the pathogenesis of DCM. As described in the section about heart insulin resistance, the initiating step is a permanent translocation of CD36 to the sarcolemma in response to chronic FFA inflow [26]. Glucagon-like peptide 1 (GLP1), the hormone of intestinal L-cells with potential cardioprotective action in DM and obesity, preventing palmitate-induced CD36 translocation and lipid accumulation by the activation of GLP1R/Akt-dependent pathway [254]. Currently, CD36 translocation is one of the more promising targets for metabolic intervention to treat the lipid-overloaded diabetic heart dysfunction [26]. CD36 trafficking to the sarcolemma promotes uncontrolled FA uptake and oxidation with reduced glucose uptake and oxidation; thus, the diabetic heart is characterized by s reduced ability to use glucose as a fuel. Additionally, hyperglycemia, regardless of plasma FFA levels, increases the influx of FAs into cardiomyocytes by the upregulation of miR-320 and the associated increase in CD36 expression [25]. The pathomechanism of DCM is closely associated with the activation of PPARα by FAs, and thus modulating the expression of genes involved in FA and glucose metabolism in the diabetic heart [31,255,256]. It was suggested that the elevated levels of PPARα may be a direct reason for the heart remodeling, which is supported by a strong negative correlation of CD36 mRNA level with left ventricular ejection fraction (LVEF) [24]. The inhibition of PPARα-mediated lipid accumulation protects the heart from lipotoxicity in DM [257,258]. Eventually, FA uptake exceeds the rate of its oxidation and lipid deposition occurs. Increased FA oxidation and lipid deposition are accompanied by ROS production, which initiate damage to cellular structures, including mitochondria [259]. The cardiac dysfunction caused by the increased FA uptake is referred to as lipotoxic cardiomyopathy. The described metabolic alterations first result in the development of insulin resistance and ultimately lead to inefficient energy production, remodeling, and deficient contractility of heart.

Inefficient energy production is the result of the aforementioned loss of metabolic flexibility in the utilization of energy substrates, occurring in the initial phase of DCM, and is associated with decreased contractility. The subsequent lipid accumulation of FAs, TAGs, CERs, and DAGs, is also attributed to the functional abnormalities of cardiomyocytes, as they induce the so-called lipoapoptosis [259]. Chronic FA overload of cardiomyocytes contributes to the ventricular dysfunction by inducing cardiomyocytes apoptosis or necrosis, cardiac hypertrophy, and deposition of fibrous tissue [23,260,261]. Similarly, cardiac dysfunctions as a result of the CD36-dependent FAs uptake were observed in age-induced cardiomyopathy in mice [262]. However, if hypertrophic cardiomyocytes are not chronically exposed to high circulating FFAs, CD36 is necessary for the sufficient ATP production. In this case, CD36-deficiency accelerates the progression of cardiac hypertrophy to HF in response to pressure overload, as a result of restricted FA supply [263,264]. It indicates that both increased and decreased FA metabolism may be harmful to the heart.

**Figure 5:**
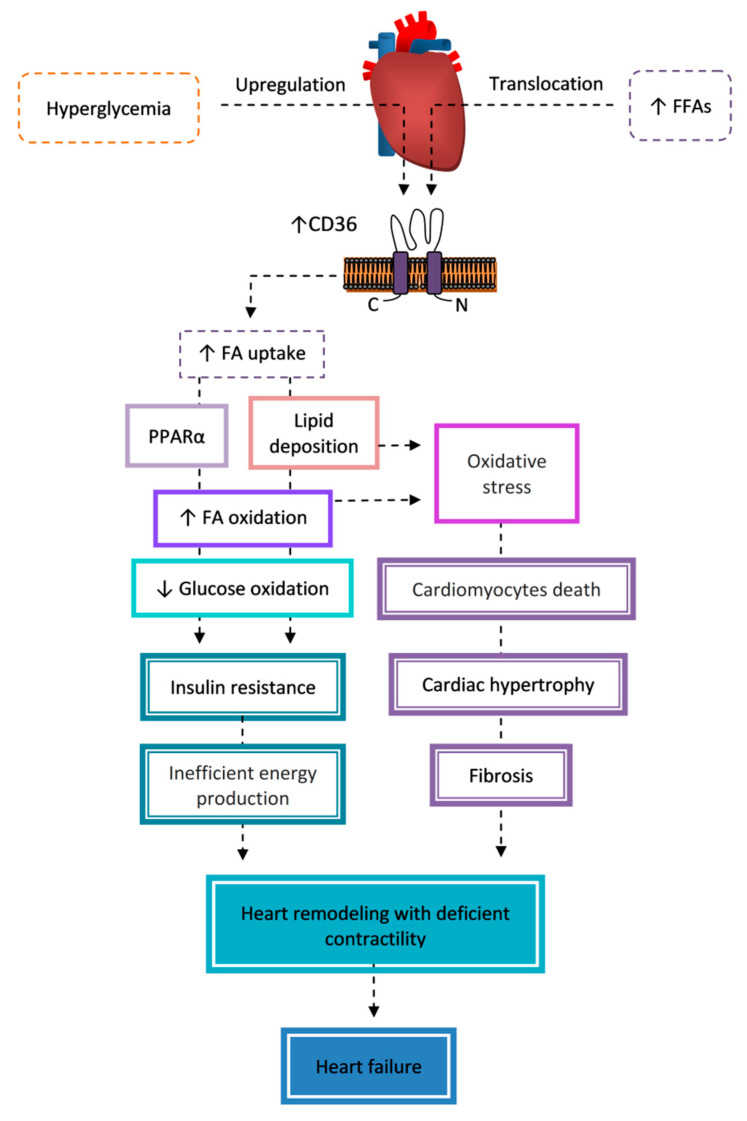
The contribution of CD36 to the pathogenesis of diabetic cardiomyopathy (DCM). The central event in the CD36-dependent mechanism of DCM pathogenesis is a persistent increase of CD36 expression in the plasma membrane, associated with the influx of fatty acids (FAs) into cardiomyocytes and lipid deposition. Some of the effects are an alteration of cardiomyocyte energy metabolism to the utility of FAs, and a decrease of glucose oxidation, which promotes ineffective energy production. The other is oxidative stress, caused by accumulated lipids and FA oxidation, stimulating cardiomyocyte death, hypertrophy, and fibrosis. They are followed by heart remodeling, deficient contractility, and ultimately heart failure.

In a situation of chronic FA overload, the inhibition of CD36 [10,23,265], or CD36-deficient [256] reduced FA uptake, leads to the promotion of glucose uptake and glycolysis, the reduction of FA oxidation, lipid accumulation, and ROS generation, thereby protecting cardiomyocytes from lipotoxicity and oxidative stress. Such endogenous substances as fibroblast growth factor 21 (FGF21) [266] and a newly identified adipokine apelin [267] limit DM-induced increase in CD36 expression, thereby reducing FA uptake and metabolism. These effects translate into the function of the heart. The results of CD36 inhibition in mice on a high-fat diet were a decrease of heart/body weight ratio, an increase of LVEF and fractional shortening, and a normalization of LV diameter [23]. These results indicate the association of CD36 with remodeling in the diabetic heart, and the CD36-dependent FA transport and metabolism are crucial to its occurrence (Figure 5).

## 8. Summary and Future Perspectives

The multifunctionality and the pattern of tissue expression of CD36 make it an important mediator not only in the physiological, but also in the pathological metabolism of lipids and carbohydrates. CD36 is necessary to maintain an adequate supply of lipids to cells, energy metabolism, and the immune response. However, alterations in the expression of CD36 and its signaling pathways in response to hyperglycemia or dyslipidemia will result in serious consequences for adipose tissue, the liver, muscles, the heart, β-cells, the kidneys, the eyes, or peripheral nerves. This makes CD36 “an enemy” rather than “a friend”.

The contribution of CD36 to the pathogenesis of DM is associated with two major pathophysiologic events—insulin resistance and impaired pancreatic β-cell function. The role of CD36 is mainly the cellular uptake of FA with the modulation of the rate of FA uptake by dynamic subcellular recycling and regulation of expression. The most sensitive to the development of insulin resistance is adipose tissue, which plays a key role in the initiation of insulin resistance in other tissues by releasing significant amounts of FFAs. The association of CD36 and reduced adipose tissue insulin sensitivity with the limitation of the inhibitory effect of insulin on lipolysis is based on inflammation, hypertrophy of adipocytes, and the adverse profile of adipokines. Excessive plasma FFAs, derived from lipolysis of adipose tissue or diet, may result in the intracellular influx of FAs exceeding the capacity of cells for their utilization. CD36 plays a key role in this process, leading to pathological accumulation of lipids (TAGs, DAGs, and CERs) in non-adipose tissue, such as liver, heart, and muscle, which initiate lipotoxicity and insulin resistance. A typical feature of the insulin resistant liver is a steatosis in which accumulating lipids antagonize insulin signaling, further elevate CD36 expression in the plasma membrane, disrupt mitochondrial function, and increase TAG accumulation. However, increased expression of CD36 in hepatocytes also stimulates the removal of FAs by VLDL secretion, but this is associated with the worsening of dyslipidemia. Skeletal and cardiac myocyte overexposures to lipids result in GLUT-4 being trapped in a non-endosomal storage compartment and translocation of CD36 to the sarcolemma associated with excessive FA transport and lipid accumulation, implying insulin signaling by decreased glucose transport and its incorporation into glycogen. Moreover, in cardiomyocytes, FA oversupply changes the expression of genes associated with FA and glucose utilization. Ultimately, these actions result in a transition to energy from FAs instead of glucose and contractile dysfunction, e.g., expressed as a decrease in cardiac efficiency. Furthermore, progressive glycemic and lipid abnormalities lead to the dysfunction and damage of β-cells. Impaired glucose-stimulated insulin secretion is an early and central event in the pathogenesis of T2DM and is already observed in prediabetes. Similarly to insulin resistance, the role of CD36 in the dysfunction of insulin secretion is associated with an increase in FA influx followed by several effects, such as CER accumulation, inflammation, decreased insulin mRNA expression, and defective insulin exocytosis. Lipid and glucose toxicity also underlie the CD36-dependent mechanism of β-cell damage, but a reduction of β-cell mass over time plays a secondary role. All these metabolic events accelerate the progression of prediabetes to DM.

The roles of CD36 in diabetes complications are different. Hyperglycemia and dyslipidemia are seen as the main causes of diabetes complications, but the profiles of the CD36-dependent pathogenic mechanisms are not the same. They become tissue-specific. In the kidney, lipid overexposure causes glomeruli damage by the induction of podocyte and mesangial cell apoptosis and sclerosis, while hyperglycemia and the products of oxidation and glycation mediate apoptosis of tubular cells or fibrosis of the interstitium, resulting in tubulointerstitial injury. These various CD36-dependent mechanisms eventually lead to the progressive loss of kidney function in diabetics. In other complications, the involvement of CD36 in the pathogenesis is much less understood. In the retina, CD36 mainly mediates microvascular cell damage in response to the overload of modified LDL or damage to photoreceptors, and RPE cells by inflammation mediated by mononuclear phagocytes or oxLDL. These events are associated with the progressive retina injury and loss of vision. On the other hand, CD36 seems to prevent neovascularization, which is characteristic of advanced retinopathy. Even less is known about the importance of CD36 in the pathogenesis of DPN. Several hypotheses have been presented that encompass the peripheral nerve response to hyperglycemia and FFAs mediated by CD36. They were expressed as inflammation, and glucose and lipid metabolism disorder accompanied by lipid accumulation. These actions appear to affect Schwann cells and cause their dysfunction, which may result in inefficient myelination and the loss of supportive action on neurons. In DCM, increased FA oxidation and lipid deposition induce oxidative stress with the damage to cardiomyocytes, followed by inefficient energy production, remodeling, deficient contractility of the heart, and ultimately HF.

Due to the still insufficient prevention of DM and the treatment of DM complications, we would like to emphasize several promising research goals based on CD36. The first is the modulation of *CD36* gene expression by PPAR—the major regulatory mechanism of gene transcription. Another target is subcellular trafficking of CD36. The relocation of CD36 is a complex mechanism, involving the v-ATPase of endosomes and VAMPs; it is regulated by many metabolic factors (hyperinsulinemia, hyperglycemia, or increased plasma FFAs), and effects the supply of FAs or modified lipoproteins to the cell that determines lipotoxicity. The mechanisms mediated by CD36, including lipotoxicity, often induce oxidative stress, leading to cell death, as is the case with podocytes, tubular cells, pericytes, and endothelial and RPE cells, but also fibrosis of the glomeruli, the interstitium, or the heart. There are attempts to reduce CD36-mediated oxidative stress.

An important part of our work was also the discussion of the utility of sCD36 in the monitoring of DM or its complications. The role of sCD36 as a DM marker has been widely discussed, but the position on this matter is still ambiguous. This is due to the association of sCD36 with T2DM risk factors, for example, metabolic syndrome, and heterogeneity of the studied populations, but also the lack of standardization of sCD36 determinations. However, it seems promising to use sCD36 as a DN marker as its level correlates with the severity of nephropathy.

CD36 therefore offers broad research perspectives in both the monitoring and treatment of DM and its complications. All the more so because it is still an inexhaustible issue and leaves many questions unanswered.
